# Dual transcriptomics suggests trophoblastic sequestration and splicing blockade induced by *Trypanosoma cruzi* during *in vitro* infection

**DOI:** 10.1128/spectrum.00887-25

**Published:** 2025-10-20

**Authors:** Tatiana M. Cáceres, Lissa Cruz-Saavedra, Luz Helena Patiño, Manuel Alfonso Patarroyo, Juan David Ramírez

**Affiliations:** 1 Centro de Investigaciones en Microbiología and Biotecnología-UR (CIMBIUR), School of Sciences and Engineering, Universidad del Rosario 25807, Bogotá, Colombia; 2 Grupo de Investigación Básica en Biología Molecular e Inmunología (GIBBMI), Fundación Instituto de Inmunología de Colombia (FIDIC) 91481, Bogotá, Colombia; 3 Microbiology Department, Faculty of Medicine, Universidad Nacional de Colombia 28021, Bogotá, Colombia; 4 Health Sciences Faculty, Universidad de Ciencias Aplicadas y Ambientales (U.D.C.A) 157898, Bogotá, Colombia; 5 Center for Global Health and Inter-Disciplinary Research, Department of Global, Environmental and Genomic Health Sciences, College of Public Health, University of South Florida 27117, Tampa, Florida, USA; Institut de recherche pour le development, Montpellier, France

**Keywords:** chagas disease, congenital transmission, molecular interactions, transcriptomics

## Abstract

Congenital transmission of *Trypanosoma cruzi* is a significant public health concern, involving complex interactions between the parasite, the placenta, and the fetus. However, the mechanisms facilitating parasite passage across the placental barrier remain incompletely understood. This study explores the transcriptional response of both *T. cruzi* (TcI) and human trophoblastic cells during *in vitro* infection, aiming to provide insights into parasite-host interactions relevant to congenital Chagas disease. Two TcI strains (MHOM/CO/01/DA and MHOM/CO/04/MG) were used to infect BeWo cells, and RNA was sequenced at 72 and 120 h post-infection. The MG strain exhibited higher invasion and replication rates than the DA strain. Transcriptomic analysis of the parasite revealed modulation of 157 genes, including several encoding surface-associated multigene families potentially involved in host interaction. In host cells, the upregulation of histones and ribonucleoproteins suggested chromatin remodeling, while the downregulation of RNA splicing-related genes pointed to alterations in RNA processing. At 120 h, genes associated with necroptosis were upregulated, indicating a shift in the host cell response. Overall, these results highlight key molecular changes during trophoblast infection by TcI strains and provide a transcriptomic resource for advancing the understanding of congenital *T. cruzi* infection.

**IMPORTANCE:**

Congenital transmission of *Trypanosoma cruzi*, the causative agent of Chagas disease, remains a significant global health concern. However, the mechanisms by which the parasite crosses the placenta and impacts fetal development are not yet fully understood. This study investigates the interaction between *T. cruzi* and trophoblastic cells—key components of the placental barrier—by analyzing transcriptomic changes in both the parasite and the host during infection. The findings provide insight into the molecular alterations associated with congenital infection and contribute to a better understanding of the host-parasite dynamics at the maternal-fetal interface. These results offer a valuable resource for advancing research into the biological processes underlying congenital Chagas disease.

## INTRODUCTION

Chagas disease (ChD), caused by the protozoan *Trypanosoma cruzi* (*T. cruzi*), remains a major public health challenge in Latin America, affecting an estimated 6–7 million people worldwide (1). *Trypanosoma cruzi* can be transmitted through various routes, including the feces of triatomine vectors, blood transfusion from infected donors, organ transplantation, ingestion of contaminated food or beverages, laboratory accidents, and congenital transmission from infected mothers to their offspring (2). Although vectorial transmission has decreased significantly thanks to effective control programs and enhanced donor screening, congenital transmission has become increasingly relevant, especially in non-endemic regions due to population migration (3). Globally, over 2 million women of childbearing age are infected with *T. cruzi*, and congenital transmission occurs in 1%–10% of pregnancies. In endemic areas of Latin America, approximately 9,000 infants are born with the infection each year, accounting for up to 22% of new cases. The lack of widespread prenatal screening and the limited availability of safe treatment options for pregnant women and neonates further exacerbate the problem, underscoring the need for improved early detection and intervention strategies (4–6).

The mechanisms by which *T. cruzi* overcomes the placental barrier and evades the immune response remain poorly understood. Transmission is believed to occur through the hematogenous route, with trophoblastic cells playing a key role in facilitating parasite migration into fetal circulation. These cells are essential for placental function, regulating proliferation, differentiation, and programmed cell death to maintain a protective barrier (7). An alternative transmission route, independent of the trophoblast, has also been described. This pathway involves the marginal zone of the placenta, which consists of non-trophoblastic epithelium and smooth muscle cells embedded in an extracellular matrix. In this region, *T. cruzi* can invade local cells and, after evading placental defense mechanisms, eventually reach myocytes and endothelial cells lining the fetal vessels within the chorionic plate or umbilical cord, ultimately gaining access to the fetal circulation (8–10).

*T. cruzi* exploits virulence factors such as Cruzipain, a protease that degrades extracellular matrix components like collagen and fibronectin while activating host matrix metalloproteases. This disruption increases trophoblast turnover, induces apoptosis via caspase 8 activation, and damages placental integrity, ultimately enhancing the risk of congenital transmission (11–13). The inflammation and trophoblast loss caused by *T. cruzi* compromise the placental barrier, increasing the risk of fetal infection, growth restriction, or pregnancy loss (14, 15). Despite significant advancements, the molecular interactions between trophoblasts and the parasite remain poorly understood, underscoring the need for further research on congenital ChD (16, 17). Transcriptomic analyses have revealed that *T. cruzi* disrupts key biological processes, including the upregulation of proteases such as MMP-2 and MMP-9, as well as innate immunity genes like CD46 and Toll-like receptors (18–20). Notably, strains isolated from cases of congenital Chagas disease downregulate cell cycle-related genes, potentially inducing trophoblast arrest, whereas highly virulent strains predominantly activate inflammatory pathways. These findings underscore the ability of *T. cruzi* to manipulate placental function in ways that may favor vertical transmission (21–24).

Understanding congenital ChD requires examining *T. cruzi* remodeling, as the parasite exhibits significant diversity in size, structure, ploidy, and genetic classification into at least six discrete typing units (DTUs) (24–27), while most studies have focused on DTUs TcII, TcV, and TcVI. DTU I—associated with variable virulence—remains largely understudied, despite congenital ChD rates reaching 4% in Colombia, 3.8% in Ecuador, and 6.5% in Venezuela (28–32). The low number of reported cases raises concerns about potential underreporting or whether DTU I possess intrinsic characteristics that limit its transmission or detection. These uncertainties underscore the need for further research on its interaction with trophoblastic cells.

The remodeling of the *T. cruzi* transcriptome during intracellular development highlights dynamic morphological and functional changes, particularly in nutrient acquisition, stress responses, and cell cycle regulation, though these processes remain incompletely characterized (33). Similar to *T. brucei* and *Leishmania donovani*, *T. cruzi* relies on mRNA stability during early development, while translational efficiency and post-translational modifications play key roles in maintaining homeostasis and adapting to the host environment (33–35). Unlike bacterial operons, *T. cruzi*’s polycistronic RNA organization does not necessarily cluster functionally related genes, adding complexity to its post-transcriptional regulation (33, 34). This underscores the significance of transcriptomics in unraveling the parasite’s molecular strategies. By integrating transcriptomic, proteomic, and metabolic data, researchers can identify key pathways involved in host recognition, immune evasion, and cellular remodeling, providing a comprehensive view of *T. cruzi*’s infection dynamics. A deeper understanding of these mechanisms could reveal novel therapeutic targets and intervention strategies.

Li and colleagues remain the only researchers to have analyzed the *T. cruzi* transcriptome during infection. Their study revealed that, during the early intracellular phase, the parasite upregulates genes encoding surface proteins such as trans-sialidases, mucins, MASPs, and gp63, as well as genes involved in flagellar assembly and motility. These findings underscore *T. cruzi*’s ability to manipulate host cellular functions to evade immune responses, highlighting the crucial role of transcriptomic studies in advancing our understanding of parasite-host interactions (33, 36, 37). Dual RNA-seq has transformed the study of *T. cruzi*-host interactions by enabling the simultaneous analysis of both organisms, providing a comprehensive view of disease progression and immune responses (38, 39). Unlike traditional transcriptomic studies that examine either the host or the pathogen in isolation, this approach captures their dynamic interplay, accounting for cell-type-specific susceptibilities and functional states (38). It also facilitates the identification of differential gene expression patterns associated with pathogenicity and virulence, which vary depending on the parasite strain and developmental stage (40, 41). By applying this methodology, this study aims to characterize the transcriptional remodeling of both *T. cruzi* (TcI) and human trophoblastic cells (BeWo) during infection, offering valuable insights into the molecular mechanisms underlying congenital transmission and infection outcomes.

## MATERIALS AND METHODS

### Parasite culture

Epimastigotes of *T. cruzi* strains MHOM/CO/01/DA (TcI) and MHOM/CO/04/MG (TcI), isolated from humans and obtained through donations to our research center, were cultured at 26°C in liver infusion tryptose (LIT) medium supplemented with 10% fetal bovine serum (FBS) and 1% penicillin-streptomycin.

### DNA extraction and *Trypanosoma cruzi* genotyping

DNA extraction was performed using 1 mL of *T. cruzi* culture at a concentration of 1 × 10⁶ parasites/mL for both the MG and DA strains, following the Dneasy Blood & Tissue Kit protocol (Qiagen, catalog No. 69504). The quality of the DNA was initially checked with a NanoDrop 2000 spectrophotometer, ensuring concentrations above 50 ng/µL and achieving 260/230 and 260/280 ratios close to 2 for optimal purity. DNA quality was also verified by 2% agarose gel electrophoresis for further confirmation.

For genotyping, PCR amplification targeted the Spliced Leader intergenic region of the mini-exon gene (SL-IR) using primers TCC (5′-CCCCCTCCCAGGCCACACTG-3′), TC1M (5′-GTGTCCGCCACCTCCTTCGGGCC-3′), and TC2 (5′-CCTGCAGGCACACGTGTGTGTG-3′); the 24S ribosomal subunit with primers D71 (5′-CCCCCTCCCAGGCCACACTG-3′) and D72 (5′-GTGTCCGCCACCTCCTTCGGGCC-3′); the 18S ribosomal subunit with primers V1 (5′-CAAGCGGCTGGGTGGTTATTCCA-3′) and V2 (5′-TTGAGGGAAGGCATGACACATGT-3′); and the cytochrome b (cytB) gene with primers P18 (5′-GACAGGATTGAGAAGCGAGAGAG-3′) and P20 (5′-CAAACCTATCACAAAAAGCATCT-3′). Sequencing was performed using the Sanger method.

Upon receiving the chromatograms, the sequences were cleaned using Unipro UGENE 50.0 to remove low-quality regions. BLASTn analysis was used to compare the sequences against a custom database built from NCBI data for each gene. For the mini-exon gene, five sequences from different DTUs with the accession numbers MF144857.1, MW477908.1, MW477921.1, and MBSY01000470.1 were used. For the 18S gene, 9 sequences from various DTUs with the accession numbers AF245382.1, AF239980.1, JN942611.1, AF301912.1, AF288660.1, AF303660.1, AJ009148.1, AF232214.1, and AF245383.1 were included. For the cytochrome *b* gene, six sequences with the accession numbers FJ549391.1, AJ130931.2, KC951604.1, FJ555648.1, JF267935.1, and AJ130935.2 were used.

The sequence analysis was performed using BLASTn with an indexed database, applying a 95% identity threshold and a minimum coverage of 80% for the alignments. An *E* value of 1*e*^−5^ was used to ensure statistical significance. Additionally, a filter was applied to remove low-complexity regions, and results were limited to the top 10 alignments per query to obtain relevant information and minimize redundancy. Genotyping was confirmed with identity scores exceeding 95% and *E* values below 1 × 10⁻⁵⁰.

### Metacyclic trypomastigote and cell-derived trypomastigote production

Metacyclic trypomastigotes were obtained from epimastigote cultures maintained in LIT medium supplemented with 10% fetal bovine serum (FBS) and 1% penicillin/streptomycin, incubated at 26  °C for 12 days at a density of 1 × 10⁸ parasites/mL. Parasite purification was performed using DEAE-Sepharose ion exchange chromatography, following the protocol described by Cruz-Saavedra et al. (42). After purification, parasites were washed with sterile, filtered 1× PBS. Viability was assessed by wet mount light microscopy, and quantification was conducted using a Neubauer chamber.

To obtain cell-derived trypomastigotes (CDTs), VERO cells were cultured to semi-confluence in 25 mL flasks with RPMI medium supplemented with 5% FBS and 1% penicillin/streptomycin. Cells were trypsinized, counted using a Neubauer chamber, and seeded at a density of 2.1 × 10⁵ cells/mL. After 24 h of adhesion, cells were infected with metacyclic trypomastigotes from the MG and DA strains at a parasite-to-cell ratio of 15:1. Cultures were monitored daily by inverted microscopy to observe intracellular amastigote nests, and the medium was replaced every 2 days. Once CDTs were detected in the supernatant, they were collected, washed with 1× PBS by centrifugation at 2,500 rpm for 10 min, and used to reinfect fresh VERO cell cultures every 5 days. This iterative process enabled CDT amplification for subsequent infection experiments in trophoblastic cell lines.

### Trophoblast infection with *T. cruzi* strains and assessment of infection

The BeWo cell line cultures, provided through a collaboration with the University of Antioquia, were seeded in 24-well plates at a density of 120,000 cells/mL. Cells were allowed to adhere and reach semi-confluence over a 2 h period in HAM-F12 medium supplemented with L-glutamine, 10% fetal bovine serum (FBS), and 1% antibiotic-antimycotic solution. Following the adhesion period, cell-derived trypomastigotes (CDTs) from two *T. cruzi* TcI strains (MG and DA) were added at a 10:1 infection ratio (trypomastigotes per cell). After 24 h of incubation, the supernatant was carefully removed, and the culture medium was replaced to maintain optimal conditions for the experiment. This multiplicity of infection (MOI) has been previously employed in our research center, as documented in the studies by Cruz et al. (43) and Cáceres et al. (44), both of which used comparable experimental conditions. Additionally, it aligns with the protocol reported by Faral and colleagues, who used an MOI of 5:1 to infect trophoblastic cells *in vitro* with *T. cruzi*, under conditions similar to those of the present study (24, 43, 44).

To assess the infection, control and infected groups were established, with evaluations conducted at specific time intervals (24, 48, 72, 96, and 120 h), as outlined in previous studies (33). Each group included three biological replicates. Infection curves were generated by quantifying the number of amastigotes per cell using Field and DAPI staining. After staining, images were captured at 40× magnification, and the number of amastigotes per cell was quantified in 300 cells using ImageJ software, with Python-based filters applied to facilitate manual counting. The number of cell-derived trypomastigotes (CDTs) was determined using a Neubauer chamber. Statistical analysis was performed using Tukey’s multiple comparison test in GraphPad Prism to identify the *T. cruzi* DTU with the highest infection potential based on the maximum number of amastigotes per cell and the highest trypomastigote count in the supernatant.

### Trophoblast infection, RNA extraction, and sequencing

For this study, DTU I was selected due to its greater performance in previous experiments and the results reported by our center, which indicated higher infectivity of this DTU in *in vitro* assays (44). Given the limited information on the ability of DTU I to infect trophoblastic cells and the broad intra-TcI diversity, both strains (MG and DA) were used to rule out potential differences in their infectivity and/or tropism. Infection assays were conducted in 25 mL culture flasks, with a cell density of 120,000 trophoblasts/mL and a ratio of 10 trypomastigotes per cell, using HAM-F12 medium supplemented with L-glutamine, 10% fetal bovine serum (FBS), and 1% antibiotic-antimycotic solution. Based on the infection curves, two specific time points were selected for further analysis: 72 h post-infection (pi), corresponding to the peak of amastigotes per cell, and 120 h pi, corresponding to the maximum number of cell-derived trypomastigotes (CDTs). Each condition was tested with three biological replicates and one technical replicate to ensure the reliability and reproducibility of the results.

RNA was extracted from both uninfected and MG-infected trophoblasts at 72- and 120 h post-infection (pi) using the RNeasy Plus Mini Kit (Qiagen), which eliminates DNA and selectively elutes RNA, following the manufacturer’s instructions. RNA concentration and integrity were assessed using NanoDrop spectrophotometry and agarose gel electrophoresis. The RNA was then sent to Novogene Bioinformatics in the United States for DualSeq library preparation and sequencing on the Illumina NOVAseq platform, generating 150 bp reads. In the control samples, 3 GB of data were obtained, while the infected cells yielded a total of 9 GB of data. This process produced 9 GB of paired-end transcriptomic data from both the parasite and the host cells, enabling comprehensive analysis of gene expression in both organisms (33). Post-sequencing, FastQC was used to assess data quality across ten parameters, including sequence quality per base, GC content, and k-mer content. No trimming was performed, as no low-quality sequences were identified. However, for the infected cell sequences, the overrepresented sequences parameter raised an alert, which may be related to the highly repetitive sequences of the parasite.

### Mapping and differential expression analysis

Transcriptomic analysis began with indexing the human reference genome GRCh38/hg38 (GCF_000001405.26, accessed in 2024) available at https://www.ncbi.nlm.nih.gov/datasets/genome/GCF_000001405.26, using Bowtie2 version 2.4.4, a widely used algorithm for fast and memory-efficient alignment of short DNA sequences. Bowtie2 enables efficient mapping of reads to a reference genome, suitable for large data sets. After indexing, alignment was performed with STAR version 2.7.10b (45), a highly efficient tool optimized for RNA-Seq data that maps reads with high sensitivity and accuracy, especially for spliced and long reads. This tool is advantageous for processing large RNA-Seq data sets in transcriptomic studies. Unmapped reads from the human genome alignment were subsequently re-mapped against the *T. cruzi* Brazil A4 TcI genome (TriTrypDB, 2024), using the same STAR alignment parameters to identify parasite-specific sequences in the RNA-Seq data. This two-step mapping ensures comprehensive gene expression profiling of both host and pathogen in mixed RNA samples (see Tables S3 and S4 at https://github.com/gimur/Dual-Transcriptomics-).

The alignment process employed the following parameters to guarantee high-quality and reliable results: --runThreadN 10 to utilize 10 CPU threads for parallel processing, --outFilterMultimapNmax 1 to allow only one unique alignment per read and exclude multimappers, --outFilterScoreMinOverLread 0.3, and --outFilterMatchNminOverLread 0.3 to retain reads with a minimum alignment score and match ratio of 30% of read length, and --twopassMode Basic to enable a two-pass alignment strategy optimizing splice junction detection. Gene quantification was enabled with --quantMode GeneCounts.

Following mapping, SAM/BAM alignment files along with corresponding GTF/GFF annotation files were processed using HTSeq-count (compatible with Python 3.8.12) (46) to generate gene count matrices. HTSeq-count assigns reads to genomic features accurately, a crucial step for reliable quantification of gene expression in RNA-Seq analyses. Differential expression analysis was conducted using DESeq2 (version ‘1.44.0’) (47), which models read counts using a negative binomial distribution to normalize data and identify statistically significant changes between conditions while accounting for biological variability. In this study, comparisons were made between trophoblast cells infected with TcI (MG strain) and uninfected controls, considering replicate variability. Genes with a fold change ≥2 or ≤ −2 and an adjusted *P*-value (padj) <0.01 were considered significantly differentially expressed and selected for further analysis.

The adjusted *P*-values correspond to the False Discovery Rate (FDR), calculated by DESeq2 using the Benjamini-Hochberg procedure to correct for multiple testing. This method controls the expected proportion of false positives among the significant genes by ranking and adjusting *P*-values accordingly. Applying a stringent threshold of padj <0.01 increases confidence in the identified differentially expressed genes, consistent with established RNA-Seq analysis standards.

Visualization of results was performed using RStudio version 2024.12.1+563 (RStudio, PBC; https://posit.co/products/open-source/rstudio/), employing volcano plots, heatmaps, MA plots, and Principal Component Analysis (PCA). These visualization tools enable identification of expression patterns and relationships among samples, providing valuable insights into the transcriptomic changes under the experimental conditions. RStudio offers a comprehensive IDE for R, facilitating advanced data analysis, statistical modeling, and generation of publication-quality graphics.

### Gene ontology, signaling pathway, and interaction networks

Differentially expressed gene IDs from the parasite were analyzed for ontological terms using EupathDB TriTrypDB (release 68, 7 May 2024) (48), which is a comprehensive resource for the genomic and functional annotation of *Trypanosoma* and other trypanosomatids. This analysis focused on molecular function, biological processes, and cellular components, as well as signaling pathways. For gene ontology analysis of the host, DAVID (Database for Annotation, Visualization, and Integrated Discovery) software (Knowledgebase v2024q2, released 5 July 2024) was used to identify enriched biological terms and pathways from the list of differentially expressed genes (DEGs). Pathway analysis was further conducted using KEGG (Kyoto Encyclopedia of Genes and Genomes, Release 111.0, 1 July 2024), KAAS (KEGG Automatic Annotation Server, version released 3 April 2015), and STRING (Search Tool for the Retrieval of Interacting Genes/Proteins, version 12.0), which are platforms for functional annotation and protein-protein interaction networks. The STRING analysis was performed with a minimum interaction score of 0.7, ensuring that only high-confidence protein interactions were considered in the network construction (49, 50).

## RESULTS

### Assessment of infection in trophoblasts

During the initial biological characterization, both strains demonstrated the ability to invade and replicate within trophoblastic cells, as reflected in the infection curves. These curves revealed the presence of infected cells from the earliest post-infection evaluation. Subsequently, both strains exhibited an exponential increase in the number of amastigotes per cell.

**Fig 1:**
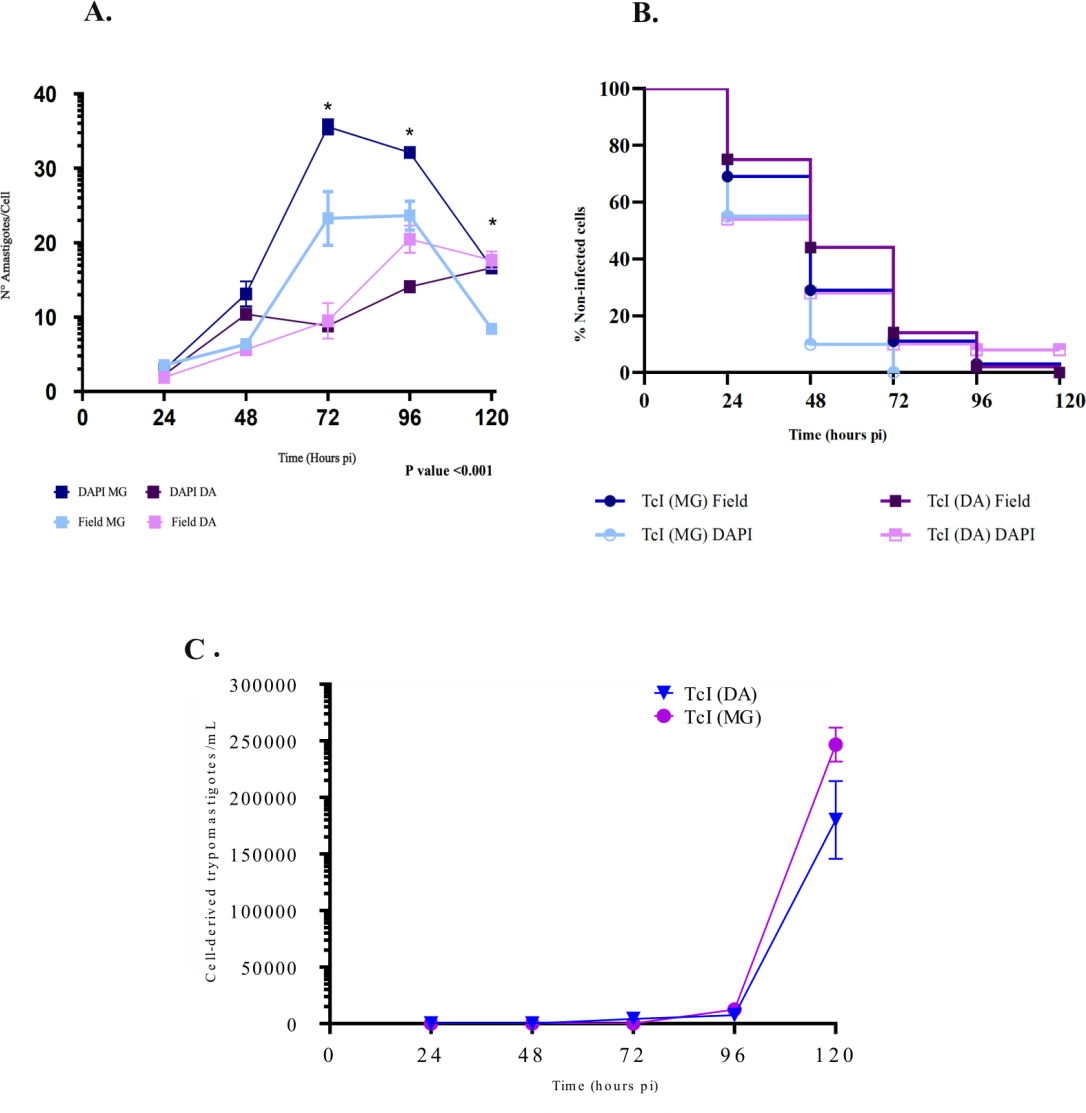
Trophoblast infection curves at 24, 48, 72, 96, and 120 h of assessment. (**A**) Number of amastigotes per cell. (**B**) Percentage of uninfected cells during the evaluated times. (**C**) Trypomastigote production derived from cells/mL.

The peak infection occurred at 72 h for the MG strain, reaching 36 amastigotes per cell, whereas the DA strain peaked at 96 h with 20 amastigotes per cell (Fig. 1A). Statistically significant differences between the strains were observed at 72 h (*P* < 0.001), 96 h (*P* < 0.001), and 120 h (*P* < 0.001), as confirmed by ANOVA (Fig. 1A).

Furthermore, the MG strain achieved 100% cell infection by 72 h, as verified by DAPI and Field staining, while the DA strain required 96 h to reach the same level (Fig. 1B; see Table S2 at https://github.com/gimur/Dual-Transcriptomics-). Regarding the production of cell-derived trypomastigotes (CDTs), although the difference between the two strains was not statistically significant, the MG strain produced a higher number of CDTs, reaching 246,666 CDTs/mL compared to 180,000 CDTs/mL for the DA strain (Fig. 1C).

### BeWo cells transcriptional landscape assessment

**Fig 2:**
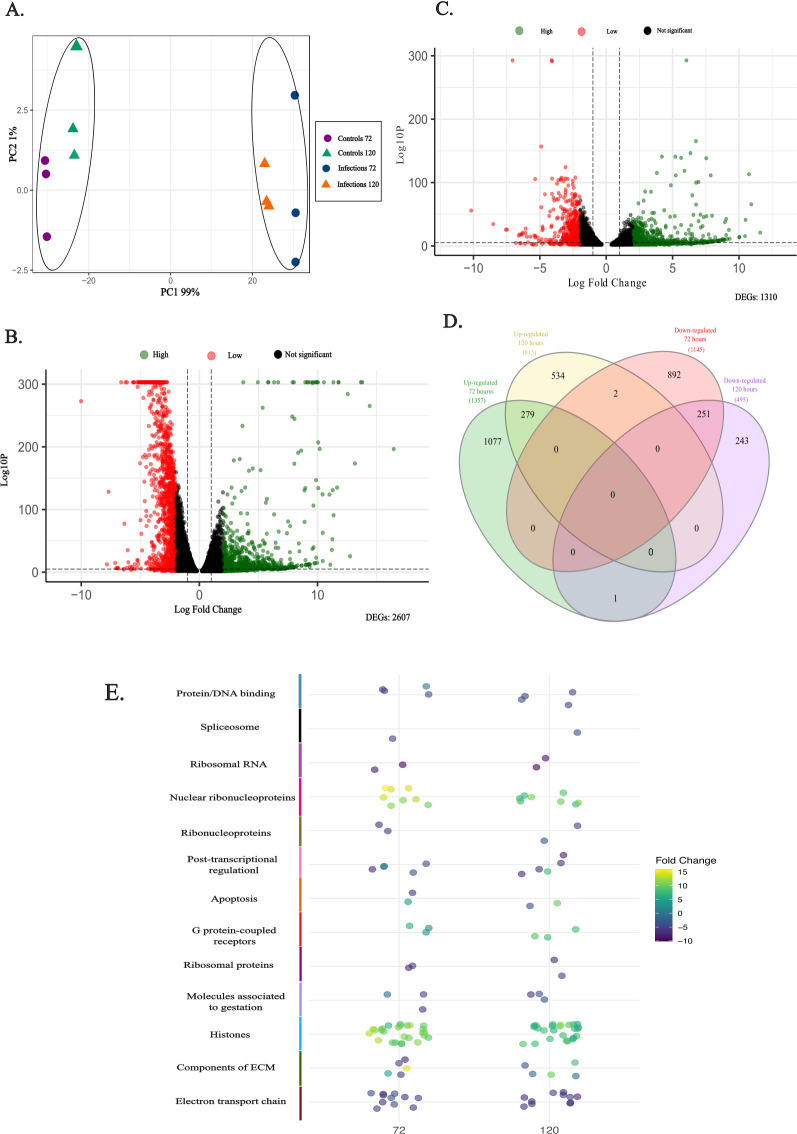
(**A**) Principal component analysis (PCA) of gene expression profiles in trophoblasts during *T. cruzi* infection. (**B**) Differential expression analysis in BeWo cells. Downregulated genes are shown in red and upregulated genes in green for two different time points: 72 h post-infection (pi) and 120 h pi. Genes were considered significantly differentially expressed based on a threshold of adjusted *P*-value (padj) < 0.01. (**C**) Venn diagram illustrating the distribution of upregulated and downregulated genes between these two time periods: in green, genes upregulated at 72 h; in yellow, genes upregulated at 120 h; in red, genes downregulated at 72 h; and in purple, genes downregulated at 120 h. (**D**) Bubble chart of genes with the highest expression changes (Fold Change) in the host at 72 and 120 h pi. The colors of the top bar correspond to the function of these genes, as specified in the conventions. (E) Bubble chart of genes with the highest expression changes (Fold Change) in the host at 72- and 120 h pi. The colors of the top bar correspond to the function of these genes, specified in the conventions.

To assess BeWo cells transcriptional remodeling, infected cells were compared to uninfected cells at two time points. Principal Component Analysis (PCA) revealed distinct clustering between the two groups, indicating significant gene expression shifts (Fig. 2A).

Differential expression analysis identified 2,607 differentially expressed genes at 72 h, with 1,357 upregulated and 1,145 downregulated. At 120 h, 1,310 genes were differentially expressed, with a predominance of upregulation (815 upregulated vs 495 downregulated) (Fig. 2B and C).

A Venn diagram showed that 279 upregulated and 251 downregulated genes were shared between both time points, suggesting an adaptive response and highlighting key genes involved in the host’s defense against infection (Fig. 2D).

During the annotation of the 50 genes with the most significant fold changes, several relevant terms were identified, some of which were shared across both time points. Notably, genes related to histones, G protein-coupled receptors, and nuclear ribonucleoproteins were significantly upregulated. Conversely, genes associated with gestation-related molecules, extracellular matrix components, DNA-binding proteins, the electron transport chain, and the spliceosome complex were downregulated (Fig. 2E).

During the gene ontology assessment, several terms identified in Fig. 2 were confirmed, along with the upregulation of genes involved in immune response, reactive oxygen species production, DNA damage response and repair, and transcriptional regulation. Additionally, increased expression was observed in key signaling pathways, including the TORC1 pathway, the MAPK cascade, cellular stress response, cell cycle regulation, and translation processes (see Fig. S1 at https://github.com/gimur/Dual-Transcriptomics-).

Analysis of the interaction network of overexpressed genes at 72 h post-infection revealed distinct functional clusters. Node 1 comprises DNA repair, chromatin structure, and genome stability genes. Notably, this node includes histone variants such as H1, H2A, H2B, H3, and H4, which are essential for chromatin organization and play critical roles in gene expression regulation, DNA replication, and repair (see Fig. S2B at https://github.com/gimur/Dual-Transcriptomics-). Node 2 consists of genes such as *ACTA2, ACTC1, ANKRD1, MYOT, NOTCH4, TTN*, and *VIM*, which are involved in cytoskeletal organization, cellular signaling, and adhesion processes, highlighting structural and functional adaptations in infected cells. Node 3 includes genes like *CYP19A1, CYP3A7*, and *HSD3B1*, which participate in the steroidogenic pathway, influencing hormone biosynthesis and metabolism. Nodes 4 and 5 contain immune-related genes, including *KLRD1* and *NCR1*, which regulate natural killer cell activity and surveillance, and *CCL28* and *CXCR6*, which are involved in chemokine signaling and immune cell recruitment to infection sites.

The spliceosome complex was consistently downregulated, prompting us to reconstruct this pathway and identify the genes within the splicing complex that were also downregulated in our study (see Fig. S1A at https://github.com/gimur/Dual-Transcriptomics-). Conversely, terms related to molecular function, oxidative stress, and apoptotic processes were significantly upregulated, suggesting an enhanced cellular response to stress and damage. In contrast, activities associated with protein binding, RNA polymerase II, and protein folding were downregulated, indicating a potential disruption in normal cellular functions.

At 120 h post-infection, apoptotic processes, particularly necroptosis, were activated. This form of programmed cell death, often triggered by inflammatory signals, may serve as a strategic response by host cells to control the infection. Our study identified upregulation of key genes, *H2AX* and *TRPM7*, associated with this pathway. *H2AX* plays a critical role in DNA repair, while *TRPM7* is involved in cellular signaling and ion homeostasis. Together, these genes illustrate the balance between cellular survival and apoptosis during infection. The interaction network of overexpressed genes in host cells at 120 h post-infection (see Fig. S3B at https://github.com/gimur/Dual-Transcriptomics-) reveals the activation of key processes organized into functional clusters. The first cluster includes genes involved in chromatin structure, gene expression regulation, DNA replication, and repair. Notable genes such as *H1-3, H2AC11, H2BC1, H3C10, H4C12, ATM, BRCA2,* and *BARD1* are essential for DNA damage response and genome stability. The second cluster consists of genes associated with transcriptional regulation and mRNA processing, including *AFF4, CCNT2, HEXIM2, PCF11, RPRD2,* and *SETX*, reflecting the modulation of transcription elongation and RNA polymerase II activity. The third cluster encompasses genes like *PKD1, PKHD1, TRPC1, TRPM6,* and *TRPM7*, which are involved in calcium signaling and ion channel regulation. These processes are vital for cellular homeostasis and signaling during infection. The upregulation of *TRPM7* highlights its role in cellular signaling and ion homeostasis, critical for the host’s response to infection. The fourth cluster features genes related to intracellular signaling pathways and phosphoinositide metabolism, including *CAMK4, DAPP1, PIKFYVE,* and *PLCG2*. These genes are integral to calcium-mediated signaling, immune cell activation, and vesicular trafficking, playing crucial roles in immune responses and cellular communication during infection.

### When infecting trophoblasts,*Trypanosoma cruzi* I alter its gene expression profile

In this study, we assessed the invasive capacity of two *T. cruzi* DTU I strains using an *in vitro* trophoblastic cell model. The MG strain exhibited significantly higher infectivity than the DA strain, evidenced by a greater number of amastigotes per cell and an increased count of CDTs (Fig. 1). These findings emphasize the biological variability within DTU I strains. Given the higher infectivity of the MG strain, we further analyzed the gene expression profiles of both the parasite and the host during MG infection.

Our analysis of DTU TcI strains revealed that the MG strain exhibited significantly greater infectivity than the DA strain *in vitro*, as evidenced by a higher production of amastigotes per cell and an increased number of CDTs (Fig. 1). These findings underscore the variability within DTUs and demonstrate how subtle biological differences can influence infection potential. Given the MG strain’s superior performance, we further investigated the gene expression profiles of both the parasite and the host during MG infection.

**Fig 3:**
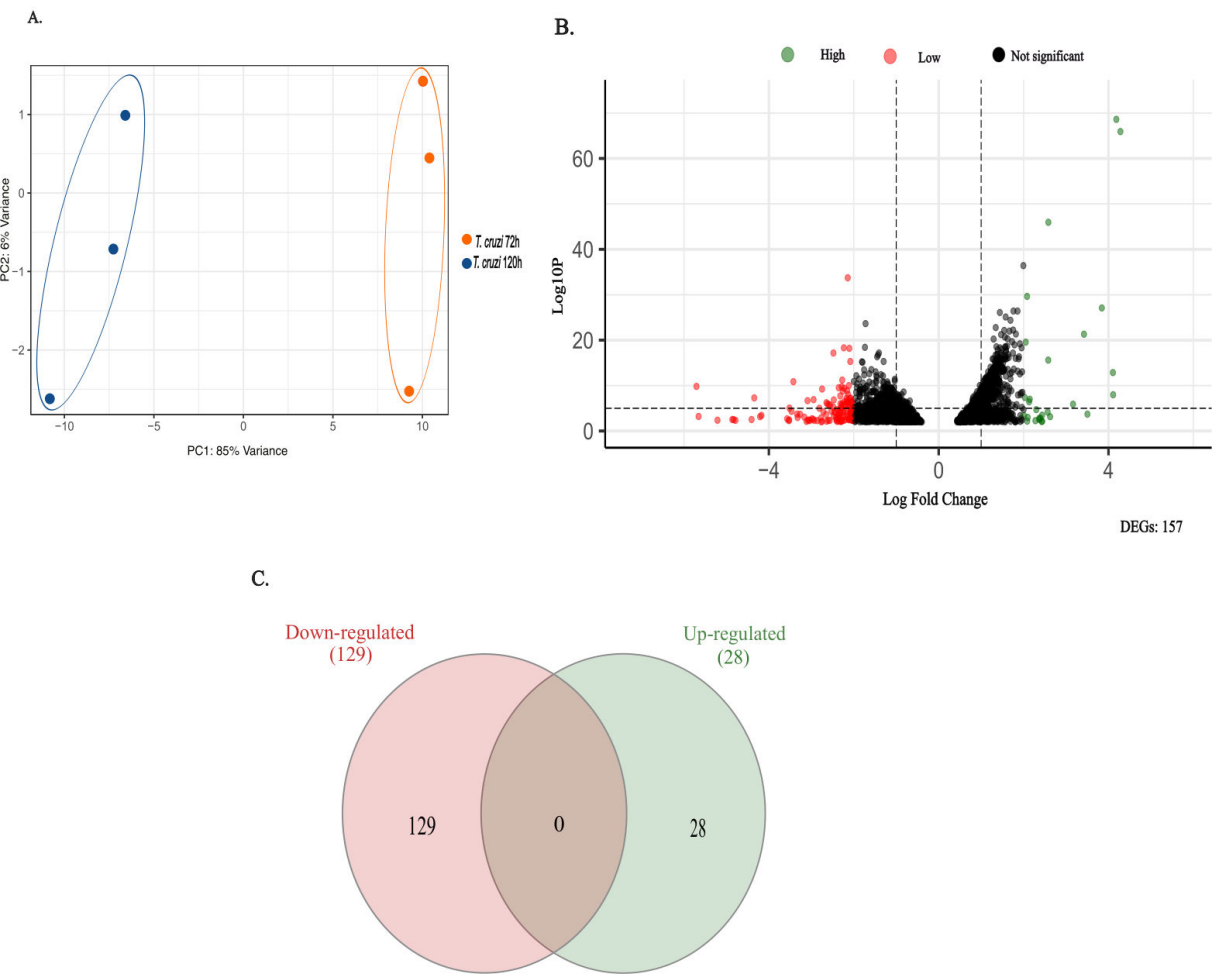
(**A**) Principal component analysis (PCA) of *Trypanosoma cruzi* during infection of trophoblastic cells. (**B**) Differential expression analysis: downregulated genes (Log Fold Change −2) are shown in red, and upregulated genes (Log Fold Change > 2) are shown in green. Genes were considered significantly differentially expressed using an adjusted *P*-value threshold (padj) < 0.01. (**C**) Venn diagram illustrating the overlap of differentially expressed genes: upregulated genes are represented in green, and downregulated genes in red.

**Fig 4:**
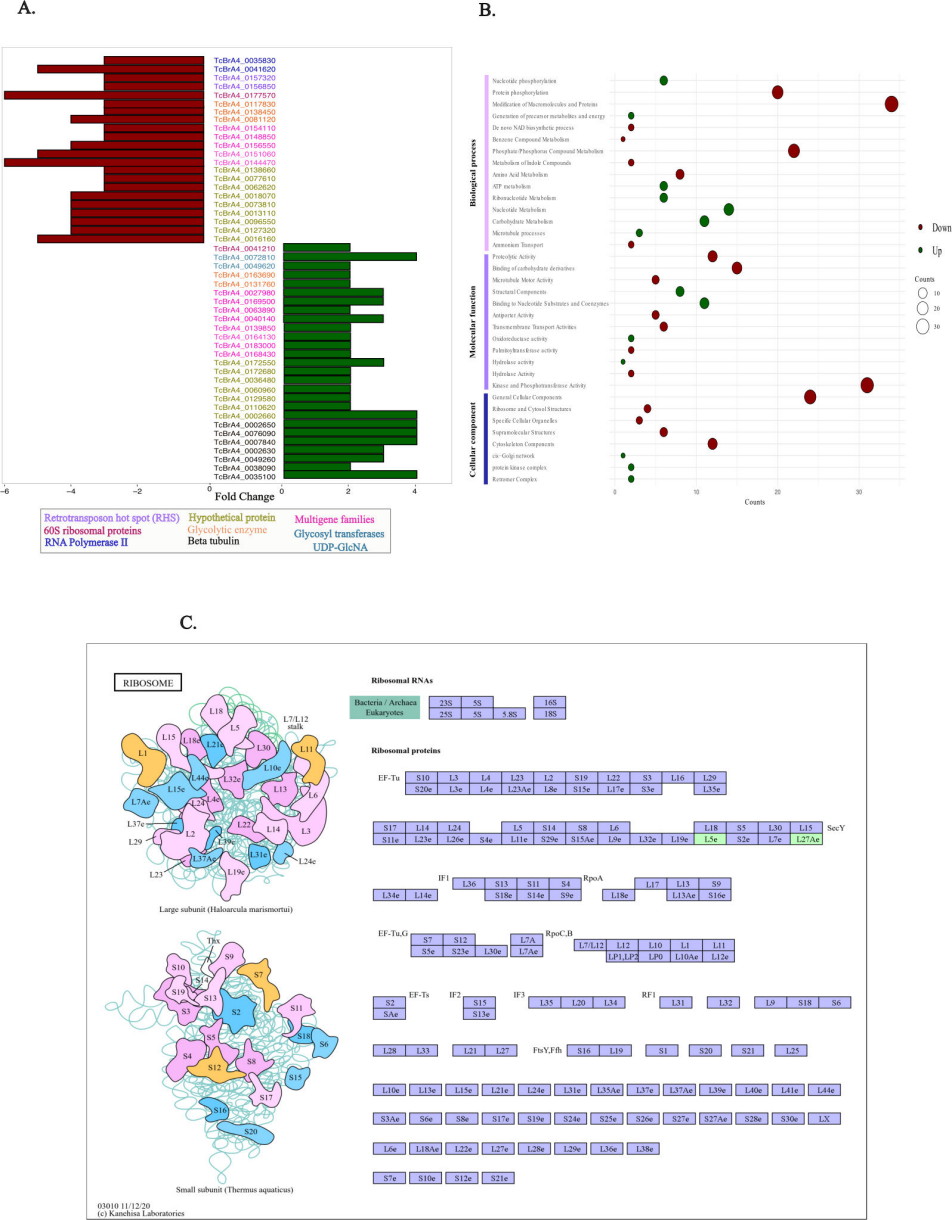
(**A**) The 50 *T. cruzi* genes showing the greatest fold change during the comparison between 72- and 120 h post-infection are displayed. The 72 h time point, corresponding to the peak of intracellular amastigotes, was used as the reference condition to evaluate transcriptional changes occurring at 120 h when trypomastigote production was highest. Upregulated genes are shown in green, and downregulated genes in red. Gene identifiers are presented in the center, with colors corresponding to their functional categories as defined in the legend. (**B**) Gene ontology analysis: upregulated genes are represented in green, and downregulated genes in red. The size of each sphere corresponds to the number of genes within each category. (**C**) On the left, the structure of the ribosome’s large subunit is shown, with the genes encoding these units represented in different colors and annotations. On the right, the upregulated pathway in *T. cruzi* identified by KAAS analysis is displayed, with genes exhibiting upregulation in our study highlighted in green.

Using a dual transcriptomic approach, we analyzed gene expression changes in *T. cruzi* at two critical stages of infection. The 72 h time point, when intracellular amastigotes peaked, was used as the reference condition, while the 120 h time point corresponded to the peak production of trypomastigotes. This comparison allowed us to capture transcriptional shifts associated with parasite differentiation and the emergence of infective forms. Principal component analysis (PCA) revealed distinct expression profiles between these stages, indicating substantial transcriptional remodeling as the infection progressed (Fig. 3A). Differential expression analysis identified 157 *T. cruzi* genes, with 28 significantly upregulated and 129 downregulated at 120 h relative to the 72 h control (Fig. 3B). No overlap was observed between the two sets, as shown by the lack of intersection in the Venn diagram (Fig. 3C). To gain insight into the biological relevance of these changes, we performed functional annotation on the top 25 upregulated and top 25 downregulated genes based on fold-change values (Fig. 4A). Additionally, gene ontology (GO) analysis was conducted for all differentially expressed genes across the three main categories—biological process, cellular component, and molecular function—offering a comprehensive view of the processes impacted. To further investigate pathway-level changes, we performed enrichment analyses using TriTrypDB and KAAS.

Among the 50 most differentially expressed genes, 13 belonged to multigene families, including trans-sialidases and mucins, though their expression patterns varied. Notably, upregulated genes included those encoding β-tubulin, glycosyltransferases, and ribosomal proteins. This trend was reinforced by GO analysis, which indicated a significant upregulation of genes involved in cytoskeletal organization and function (Fig. 4A).

In contrast, downregulated genes prominently featured RHS retrotransposons, as well as genes associated with RNA polymerase II activity and glycolytic enzymes, suggesting a potential suppression of transcriptional machinery and metabolic pathways. These findings underscore the complex regulatory dynamics of *T. cruzi* during infection, reflecting both structural adaptations and metabolic shifts essential for parasite survival and host interaction.

The gene ontology analysis identified terms related to the remodeling of cytoskeletal components and the metabolism of nucleotides, nucleosides, and energy precursors such as carbohydrates. In contrast, pathways associated with proteins, amino acids, and phosphorylated compounds were downregulated, indicating significant metabolic shifts within the cell. These changes suggest a strategic prioritization of energy production and adaptation to the parasite’s stage transition (Fig. 4B). Additionally, KAAS enrichment analysis revealed the overexpression of genes encoding ribosomal proteins, including L5e and L27Ae, which were linked to the ribosome pathway (Fig. 4C).

This study’s annotation of differentially expressed genes (DEGs) identified multigene family members, consistent with the Top 50 previously described. Among the downregulated genes, those belonging to the trans-sialidase family were predominant, reinforcing their well-documented role in parasite-host interactions. Specifically, four genes from group II (*TcBrA4_0144280, TcBrA4_0144470, TcBrA4_0148820,* and *TcBrA4_0148850*) and one gene from family VIII (*TcBrA4_0146750*) were identified.

## DISCUSSION

Congenital *T. cruzi* infection is a global health concern influenced by multiple factors, including the parasite, the mother, the placenta, and the fetus. While DTUs TcII, V, and VI have been the primary focus of congenital transmission studies due to their prevalence in highly endemic regions, recent evidence indicates that TcI can also be transmitted congenitally (7, 24–27, 51). This finding, along with reports of congenital TcI transmission in animal models such as guinea pigs and mice, as well as epidemiological studies confirming its presence in pregnant women and their newborns, expands our understanding of TcI’s role in vertical transmission (26, 52–57). These data underscore the importance of including TcI in preventive research and intervention strategies, given its significant contribution to congenital *T. cruzi* transmission.

This study assessed the *in vitro* infectivity of two *T. cruzi* TcI strains (MG and DA) in human trophoblastic cells, revealing significant differences between them. The MG strain demonstrated a higher capacity for invasion, replication, and CDT production, highlighting the importance of isolating and biologically characterizing different strains to better understand their behavior, infectious potential, and clinical implications (52, 55, 58). The observed *in vitro* differences suggest that genetic factors may influence *T. cruzi* strain behavior. Structural genomic heterogeneity among strains has been reported in the literature, potentially impacting parasite pathogenicity and providing adaptive advantages. Notable variations in antigenic composition, growth rates, and virulence have been observed even among clones derived from the same parental strain (59–62). The extensive variability within TcI complicates efforts to determine how genetic diversity within this DTU influences disease presentation (60). A major limitation of this study is the lack of genomic characterization, which could provide deeper insights into strain-specific differences. Previous studies have associated chromosome copy number variations and gene dosage in key biological processes with genomic diversity. In trypanosomatids, aneuploidy—although typically deleterious in other eukaryotes—may contribute to species-specific adaptations. These adaptations often involve the expansion of multi-gene families encoding surface proteins that play essential roles in host cell interaction, invasion, and immune evasion. The diversification of these gene families likely underlies strain variability and may help explain differences in infection dynamics (61, 63, 64). Furthermore, we acknowledge the absence of orthogonal validation methods (e.g., RT-qPCR or Western blot) for the RNA-seq data as a limitation (65). Although the transcriptomic results are consistent and methodologically sound, such validation would have further reinforced the robustness of our conclusions.

Advancements in omic technologies, particularly genomics and transcriptomics, have greatly expanded our understanding of *T. cruzi* biology, host interactions, virulence, and drug mechanisms (33, 66). This study focuses on DTU I, specifically investigating molecular interactions in the context of congenital Chagas disease, with an emphasis on the highly infectious MG strain. Our findings reveal a significant reduction in gene expression between 72- and 120 h post-infection, with 129 genes downregulated and only 28 upregulated. Among the 50 most differentially expressed genes—comprising the 25 most upregulated and 25 most downregulated—16 encode hypothetical proteins with unknown functions. These results underscore the urgent need for precise genome annotations to identify potential therapeutic targets and gain deeper insights into the parasite’s regulatory mechanisms (67). Notable variations were observed in multi-gene families encoding surface proteins, particularly mucins and trans-sialidases, with no overlapping genes between the two evaluated time points. These time points coincide with the peak of amastigote production and the subsequent release of trypomastigotes, suggesting that parasite stage transitions may drive shifts in the gene repertoire of these families. Unlike *T. rangeli*, a non-pathogenic parasite that undergoes extensive gene conversion leading to a massive reduction in multi-gene family diversity, *T. cruzi* does not exhibit similar homogenization (63, 68, 69). Instead, its ability to dynamically modulate its gene repertoire across life cycle stages may be crucial for its survival and pathogenicity within the host (63, 70).

*T. cruzi* belongs to a diverse group of unicellular organisms capable of modifying host cells to its advantage. A key component of its immune evasion strategy involves trans-sialidases, a major virulence factor of the parasite (71). The *T. cruzi* trans-sialidase (TS) superfamily comprises 1,430 genes, including 15 with enzymatic activity, over 700 inactive variants that retain the catalytic domain, and the remainder classified as pseudogenes. Genomic analyses categorize these enzymes into eight groups, with active TSs in group I and inactive TSs spanning groups II to VIII (71, 72). Our study revealed a downregulation of group II trans-sialidases, which play a crucial role in host interaction, particularly in cell adhesion and invasion (63). These proteins are predominantly expressed in metacyclic trypomastigotes and intracellular amastigotes. We propose that their downregulation is associated with the transition from amastigotes to cell-derived trypomastigotes, suggesting stage-specific regulation of these genes according to the parasite’s needs. While trans-sialidases are present across different stages, their repertoire and function vary significantly. Previous studies indicate that ASP-1 and ASP-2 are predominantly expressed in amastigotes, where they contribute to intracellular survival and immune evasion (73, 74). In contrast, TSA-1, Tc85, and SA85 are primarily expressed in trypomastigotes, facilitating host membrane adhesion and cell invasion (74–77). These findings highlight the crucial role of trans-sialidase regulation in *T. cruzi*’s adaptation and infectious capacity, underscoring its ability to fine-tune gene expression in response to distinct life cycle stages.

An upregulation of the *L5* and *L27Ae* genes, which encode ribosomal subunits, was observed, suggesting an increase in protein production essential for parasite growth and replication. Beyond their role in protein synthesis, ribosomal proteins also regulate mRNA stability and translation, underscoring their significance in modulating gene expression throughout the parasite’s life cycle. This differential expression has been documented in both *T. cruzi* and *T. brucei*, highlighting the role of ribosomal proteins as key regulators of parasite differentiation. The observed enrichment reflects the parasite’s adaptation to enhance protein synthesis, which is crucial for its survival and proliferation (78–80). Moreover, distinct expression profiles of ribosomal protein-encoding genes have been identified across different parasite stages, suggesting the presence of specialized ribosomes capable of modulating translational activity in response to various stimuli, including developmental stage, environmental conditions, and host interactions (81, 82).

Additionally, our study identified an upregulation of glycosyltransferase genes in *T. cruzi* (Fig. 4A), particularly those involved in *O*-glycosylation, a process critical for parasite virulence and host interactions. *O*-glycosylation has been linked to the production of circulating detectable antigens (CDTs), indicating its enzymatic and non-enzymatic roles in CDT differentiation (83–85). Investigating these glycosyltransferase genes may provide valuable insights into *T. cruzi* infectivity and survival, particularly in understanding how *O*-glycosylation influences the stability and function of Tc-mucins, key molecules in parasite adhesion and immune evasion (85).

The annotation revealed an upregulation of essential genes, including β-tubulin, a key component in cytoskeletal remodeling and cell división (86). β-Tubulin has been linked to the differentiation of *T. cruzi* and *Leishmania* species, underscoring its importance in parasite adaptation and survival (87). While the interaction between pathogens and the extracellular matrix is well-documented, the signaling pathways activated within the parasite remain poorly understood. Additionally, the cytoskeleton plays a role in signal transduction during host cell adhesion, yet the regulatory mechanisms involving tubulin during infection are not well characterized. These gaps highlight critical areas for future research (88).

The analysis also revealed an upregulation of nucleotide and nucleoside metabolism, along with energy precursor metabolism, while protein and amino acid synthesis were downregulated. This metabolic shift suggests a strategic reprogramming essential for parasite virulence, as intracellular amastigotes must adapt to a glucose-limited environment by relying on amino acids for energy production (89). The metabolic plasticity of *T. cruzi* is fundamental to its life cycle and transmission, enabling its survival across diverse host environments. To counteract oxidative stress, *T. cruzi* utilizes the pentose phosphate pathway (PPP) to generate NADPH, which is crucial for antioxidant defense and cellular homeostasis. This metabolic adaptation might enhance the parasite’s survival within the placental environment and facilitate its migration into fetal blood vessels. Such mechanisms are critical across multiple life cycle stages and might play a significant role in congenital transmission.

The downregulation of genes associated with retrotransposon hotspots (RHS) and RNA polymerase II activity suggests a potential impact on genomic stability and transcriptional regulation during *T. cruzi* infection. RHS elements have been proposed as drivers of genetic diversity within multigene families by utilizing pseudogenes, promoting recombination, and increasing genomic variability—key mechanisms that aid the parasite in adapting to and evading the host immune response (90, 91). This genetic diversification enables *T. cruzi* to modify its antigenic surface, enhancing immune evasion, a crucial factor in congenital transmission. Understanding the mechanisms governing transmission capacity and virulence across *T. cruzi* strains is essential for identifying potential therapeutic targets to prevent disease transmission.

*T. cruzi* undergoes extensive remodeling at multiple levels, modulating biological processes through metabolic shifts that likely serve as adaptive strategies to ensure survival under conditions of nutritional stress. These metabolic adjustments not only enable the parasite to complete its life cycle but may also enhance its persistence within the host. Two key mechanisms appear to contribute to immune evasion: variability in multigene family expression and genetic diversification mediated by RHS elements. While differences in the expression of these families during stage transitions have been documented, their precise role in congenital Chagas disease, particularly in *T. cruzi* transcriptomics, remains poorly understood. Furthermore, the role of recombination hotspots in generating genetic variability has not been sufficiently explored in this context, highlighting a promising avenue for future research. Most studies have predominantly focused on the host response, leaving a significant gap in understanding the parasite’s regulatory mechanisms. To bridge this gap, future research should investigate cytoskeleton-mediated signaling pathways and the role of RHS elements in driving phenotypic variability. These aspects are critical for developing effective control strategies and expanding our knowledge of *T. cruzi* evolution and adaptation.

Understanding how *T. cruzi* modulates gene expression in BeWo cells is essential for deciphering host-pathogen interactions, as these modifications are crucial for the parasite’s survival and replication. In this study, we examined the transcriptional remodeling of trophoblastic cells at 72- and 120 h post-infection (hpi) to assess the impact of *T. cruzi* infection. Our analysis revealed a consistent upregulation of key genes, including those encoding histones and ribonucleoproteins, suggesting an adaptive host response that may influence immune dynamics and parasite persistence (Fig. 2E). The upregulation of histone-related genes points to potential chromatin reorganization, which could alter DNA accessibility and the transcription of genes critical for the host’s response. Notably, *T. cruzi* migrates toward the host cell nucleus during its intracellular cycle, indicating a possible interaction with nuclear processes (92). Although the reasons for this nuclear localization remain unclear, evidence suggests that the parasite disrupts core metabolic pathways, potentially influencing nuclear organization and gene expression (93). Additionally, this transcriptional regulation may be associated with the accumulation of DNA damage induced by metabolic activity and oxidative stress. The observed increase in processes related to superoxide anion production and DNA repair suggests the activation of repair pathways in response to infection-induced genomic damage (94). *T. cruzi* has been shown to manipulate host DNA damage and repair mechanisms, enhancing its survival and persistence under hostile conditions. In eukaryotic cells, genomic integrity is maintained through interconnected repair networks that operate in three stages: damage recognition, signal amplification, and repair (95–97). These findings highlight the intricate molecular interactions regulating both immune responses and genomic stability during *T. cruzi* invasion and infection establishment.

While the specific upregulation of ribonucleoproteins during *T. cruzi* infection has not been extensively documented, studies have linked increased levels of these proteins to cancer-related processes involving cell cycle dysregulation and potential cell cycle arrest. Ribonucleoproteins play key roles in RNA processing and cellular stress responses, which are frequently altered in cancer cells (98). Additionally, ribonucleoproteins associated with the BBOX1-AS1/hnRNPK/GADD45A axis have been identified in chorionic villi and the serum of patients with recurrent gestational loss. According to Li et al., the upregulation of components within this pathway suggests their potential as markers of cellular dysfunction in trophoblasts. These proteins not only inhibit proliferation, migration, and invasion but also induce trophoblast apoptosis (99). In this study, we observed a downregulation of apoptotic genes at 72 h post-infection, suggesting that *T. cruzi* manipulates host cellular processes to evade immune defenses. By disrupting protective mechanisms such as trophoblast turnover, the parasite may enhance its ability to breach barriers and establish infection. Identifying the specific genes and proteins involved in this process could lead to potential therapeutic targets aimed at mitigating the parasite’s impact and preserving tissue homeostasis.

Furthermore, *T. cruzi* infection led to the downregulation of splicing-related genes, which may disrupt mature RNA production and impair RNA polymerase II function. This disruption could alter the host cell’s transcription and splicing machinery, as the parasite appears to interfere with RNA polymerase II and sequester the splicing factor U2AF35. Such mechanisms may compromise essential cellular processes, aiding the parasite in immune evasion and infection establishment (93). Investigating these molecular interactions is crucial for understanding how *T. cruzi* manipulates host cellular machinery, allowing it to persist and adapt within the host.

Additionally, we observed a downregulation of ribosomal proteins, which could impact ribosome biogenesis and fundamental processes such as cell proliferation and survival. Beyond their role in translation, ribosomal proteins contribute to critical extra-ribosomal functions, including DNA repair, apoptosis, and chemoresistance (100). Their dysregulation may induce ribosomal stress, compromising cellular responses to infection and leading to apoptotic activation and ATP level alterations, as seen in necrotic cells. These findings underscore the importance of ribosomal proteins in maintaining cellular homeostasis and highlight their potential as biomarkers of cellular dysfunction.

At 120 h post-infection, we observed an upregulation of the necroptosis pathway, adding a crucial layer of complexity to the host cell’s response to *T. cruzi* infection (ee Fig. S3A at https://github.com/gimur/Dual-Transcriptomics-). Within the interaction network of these upregulated genes, histones and calcium channels were prominently overexpressed, highlighting the intricate molecular processes activated in host cells. These findings provide valuable insights into the host’s adaptive mechanisms, emphasizing the interconnected roles of DNA repair, transcriptional regulation, ion signaling, and immune responses.

The interaction network further reveals the activation of multiple functional clusters in response to infection. One cluster involves chromatin structure and dynamics, including genes such as *H1-3, H2AC11, H2BC1, H3C10,* and *H4C12*, as well as key DNA damage response genes like *ATM, BRCA2,* and *BARD1*, which are essential for genome stability. Another cluster is associated with transcriptional regulation and mRNA processing, featuring genes such as *AFF4, CCNT2, HEXIM2, PCF11, RPRD2,* and *SETX*, which modulate transcription elongation and RNA polymerase II activity. Additionally, genes involved in calcium signaling and ion channel regulation—*PKD1, PKHD1, TRPC1, TRPM6,* and *TRPM7*—highlight the importance of cellular homeostasis and signaling during infection. These interconnected pathways underscore the host cell’s coordinated response, balancing immune activation, cellular repair, and necroptosis.

Key genes such as *H2AX* and *TRPM7* were identified as central players in the necroptosis pathway. *H2AX*, a histone variant, is crucial for DNA damage repair, while *TRPM7*, a voltage-sensitive cation channel, regulates cellular signaling and ion homeostasis (101, 102). Their upregulation suggests that necroptosis may serve as an alternative to apoptosis in the host. Necroptosis, a form of programmed cell death triggered by inflammatory signals, is involved in pathogen detection and tissue repair. However, it can also drive the production of pro-inflammatory molecules, potentially disrupting tissue integrity and immune balance (101). While most studies on *T. cruzi* infections have focused on apoptosis via the caspase-3 pathway in congenital models (15), our findings suggest that necroptosis may play an underexplored role in host-pathogen interactions. Given its dual role in pathogen clearance and inflammation, further investigation into necroptosis in *T. cruzi* infections could provide deeper insights into host tissue responses and immune regulation.

Our findings are particularly significant, as necroptosis has not been previously reported in the context of congenital Chagas disease. This pathway may represent a distinct mechanism in *T. cruzi* infections, potentially explaining why *T. cruzi* DTU I is less frequently associated with congenital transmission. Previous studies have suggested that one of the parasite’s key persistence strategies is to evade immune responses that could compromise the placental barrier. Faral et al. highlighted the parasite’s specialization in congenital isolates, emphasizing its ability to manipulate host immune responses, particularly in these cases (24). The activation of necroptosis in our study suggests a dual role for this pathway: while it disrupts cellular integrity, it also promotes the release of pro-inflammatory cytokines. This inflammatory response could exacerbate tissue damage, leading to trophoblast detachment and impairing the parasite’s ability to complete its life cycle and persist within the tissue.

This study reveals profound gene expression changes in BeWo cells during infection with *T. cruzi* TcI-MG, offering new insights into the cellular mechanisms underlying congenital Chagas disease. While the host attempts to preserve structural and genomic integrity, the parasite actively manipulates key pathways, including those regulating apoptosis. This manipulation likely represents a strategic mechanism that supports parasite survival and dissemination. Notably, the downregulation of apoptotic genes in trophoblastic cells at 72 h post-infection suggests that *T. cruzi* TcI-MG suppresses cell death to evade immune responses and disrupt normal cellular functions. By interfering with protective processes such as trophoblast turnover, the parasite may enhance its capacity to cross placental barriers and establish infection.

Additionally, the study highlights the downregulation of splicing-related genes, which could impair RNA processing and affect the host’s transcriptional machinery, further facilitating the parasite’s manipulation of host cell functions. Notably, this research uncovers previously unrecognized processes in congenital Chagas disease, shedding light on the intricate host-pathogen interactions that drive disease progression. The newly identified roles of ribonucleoproteins and ribosomal proteins in cellular dysfunction could serve as potential biomarkers for early detection and prognosis, opening avenues for future investigations into therapeutic interventions.

It is important to emphasize that our results reinforce the pivotal role of the trophoblast in the interaction with *T. cruzi*. Nevertheless, the parasite may also exploit alternative entry routes, such as the marginal zone of the placenta. By revealing the dysregulation of critical processes in trophoblastic cells, including apoptosis and cellular integrity maintenance, this study indirectly suggests a potential weakening of the placenta’s primary defense barrier. This weakening could enable the parasite to activate secondary dissemination pathways.

Consequently, a thorough understanding of both trophoblast-dependent and trophoblast-independent mechanisms is essential for developing comprehensive prevention strategies that address the multiple routes *T. cruzi* uses to access the fetal circulation. Future research should investigate the relationship between the transcriptomic alterations observed in this *in vitro* model and the structural and functional changes in the placenta in *ex vivo* or *in vivo* systems, with a particular focus on the various anatomical regions implicated in congenital transmission.

### Conclusions

Investigating *Trypanosoma cruzi* DTU I in the context of congenital Chagas disease is particularly relevant in regions where this DTU predominates. Characterizing differences in infectivity and virulence among strains enhances our understanding of parasite behavior during congenital transmission. The transcriptional remodeling observed in *T. cruzi* reveals alterations in multiple cellular processes, pointing to a complex regulatory network that may support the parasite’s adaptation to different environments and stages of its life cycle. These modulations could also influence host-parasite interactions and immune evasion. Additionally, this study identifies significant transcriptomic changes in trophoblastic cells, including alterations in gene expression related to transcriptional regulation, cell signaling, and genome organization. These findings offer insight into how *T. cruzi* may modify host cellular pathways to persist within the placental environment and contribute to congenital transmission. Overall, the data provide a valuable foundation for further exploration of the molecular mechanisms involved in host-parasite dynamics during congenital infection.
